# Supplementary material for: Genomic insights into methicillin-resistant Staphylococcus pseudintermedius isolates from dogs and humans of the same sequence types reveals diversity in prophages and pathogenicity islands
Source: PLoS One. 2021 Jul 22;16(7):e0254382. doi: 10.1371/journal.pone.0254382 (PMC8297860; doi:10.1371/journal.pone.0254382)
Supplement: S1 Table — (PDF) [file pone.0254382.s001.pdf]

Supporting information

S1 Table. Primer lists used in this study

Gene	Prophage and PI	Primer name	Oligonucleotide sequence (5’-3’)	Product size (bp)
int-1	VB16-Pro1	int1-F	TGACCACTCCACCGAAAGTG	258
		int1-R	TCACCGAATCTCCCTCCTGT	
int-2	AH18-Pro1	int2-F	GAGGTAGTAAGCCGTCACGT	552
		int1-R	TGACACGTTGACCTTGCTCT	
int-3	VB88-Pro2	int3-F	AGACAATAGTGACCTGCGGC	486
		int1-R	GGCCGAGGGTATATTGACCG	
int-4	VB16-Pro3	int4-F	ACGAACAAAGTTTACGCGGC	404
		int1-R	TTTTTGAGCAGCTGCACGTC	
int-5	AI14-Pro1	int5-F	GGCGCTTGCAACTACGTAAC	257
		int1-R	CACATGTCCTACACGCTCCA	
int-6	AK9-Pro1	int6-F	AGACACGTTTGGCAGAATTGA	492
		int1-R	TTACGTGGCCGCACATACTT	
int-7	AP20-PI1	int7-F	ACGGAACAATACACTGGCGT	413
		int1-R	CATTTGCTCGGTTACGTGGC	
int-8	AP20-PI2	int8-F	GACGGGGCTTCAAGACTGAA	522
		int1-R	CGCCACGTCTAAAGCCAGTA	
spsO	VB88-ST45	spsO-F	GCGCCGATCCTACAGTAGTT	850
		spsO-R	GGGGCTATTGCGATGTCGAT	
