# Supplementary material for: Genomic insights into methicillin-resistant Staphylococcus pseudintermedius isolates from dogs and humans of the same sequence types reveals diversity in prophages and pathogenicity islands
Source: PLoS One. 2021 Jul 22;16(7):e0254382. doi: 10.1371/journal.pone.0254382 (PMC8297860; doi:10.1371/journal.pone.0254382)
Supplement: S2 Table — (PDF) [file pone.0254382.s002.pdf]

Supporting information

S2 Table. Unique genes of human isolates predicted from Roary

Gene name	Putative function	Size (bp)	Location		
			VB88-ST45	VB16- ST112	AP20-ST181
integrase	Phage integrase	1052	φ VB88-Pro1	φ VB16-Pro1	φ AP20-Pro1
amidase	Phage lysin, N-acetylmuramoyl-L-alanine amidase	1460	φ VB88-Pro1	φ VB16-Pro1	φ AP20-Pro1
	Phage protein	249	φ VB88-Pro1	φ VB16-Pro1	φ AP20-Pro1
hypothetical protein	-	167	φ VB88-Pro2	φ VB16-Pro3	φ AP20-Pro1
hypothetical protein	-	177	φ VB88-Pro2	φ VB16-Pro2	φ AP20-Pro1
hypothetical protein	-	152	φ VB88-Pro2	φ VB16-Pro1	φ AP20-Pro1
hypothetical protein	-	350	φ VB88-Pro2	φ VB16-Pro1	φ AP20-Pro1
hypothetical protein	-	335	φ VB88-Pro2	φ VB16-Pro1	φ AP20-Pro1
spsJ	LPXTG-motif cell wall anchor domain protein	VB88-ST45=3,660<br>VB16- ST112=3,660<br>AP20-ST181=3,179			
