# Supplementary material for: Genomic insights into methicillin-resistant Staphylococcus pseudintermedius isolates from dogs and humans of the same sequence types reveals diversity in prophages and pathogenicity islands
Source: PLoS One. 2021 Jul 22;16(7):e0254382. doi: 10.1371/journal.pone.0254382 (PMC8297860; doi:10.1371/journal.pone.0254382)
Supplement: S3 Table — (PDF) [file pone.0254382.s003.pdf]

# Supporting information

**S3 Table.** Information of prophages found in this study

| Prophage | Size (bp) | %GC content | Putative *att* sequence (no.) | Integration site (Left) | (right) |
|---|---|---|---|---|---|
| φ VB88-Pro1 | 51,755 | 35.1 | CATATCAA (8) | Phosphoglycerate kinase | tRNA cluster |
| φ VB88-Pro2 | 40,400 | 35.8 | TGATACCGTTTT (12) [a] | Fe-S cluster, SufB | Protease |
| φ AK9-Pro1 | 116,327 | 32.3 | TATCTTTCTACTTCTACCATGAC AAATGCTTCAATAATATCGCCTT CTTTAATATCATTGAATTTCGCTA CTGTGATACCACATTCGTAACCTT GTGCAACTTCTTTCACATCGTCTT TGAAACGTT (127) | Hypothetical protein | Translation initiation factor 2 (IF-2) |
| φ VB16-Pro1 | 48,449 | 36.6 | N/A[b] | tRNA cluster | Phosphoglycerate kinase |
| φ VB16-Pro2 | 42,902 | 35.8 | AAAACGGTATCA (12) [a] | Protease | Fe-S cluster, SufB |
| φ VB16-Pro3 | 42,674 | 37.1 | N/A[b] | Putative sodium:sulfate symporter | Putative sodium:sulfate symporter |
| φ AI14-Pro1 | 45,039 | 34.1 | GGGCAAAAAAAGGGCAG (17) [a] | Late competence protein ComGA | Late competence protein ComGA |
| φ AP20-Pro1 | 48,202 | 35.2 | TTGATATG (8) | tRNA cluster | Phosphoglycerate kinase |

[a] *att* sequence predicted from PHASTER; [b] N/A, not available
