# Supplementary figures and images for: Genomic insights into methicillin-resistant Staphylococcus pseudintermedius isolates from dogs and humans of the same sequence types reveals diversity in prophages and pathogenicity islands
Source: PLoS One. 2021 Jul 22;16(7):e0254382. doi: 10.1371/journal.pone.0254382 (PMC8297860; doi:10.1371/journal.pone.0254382)

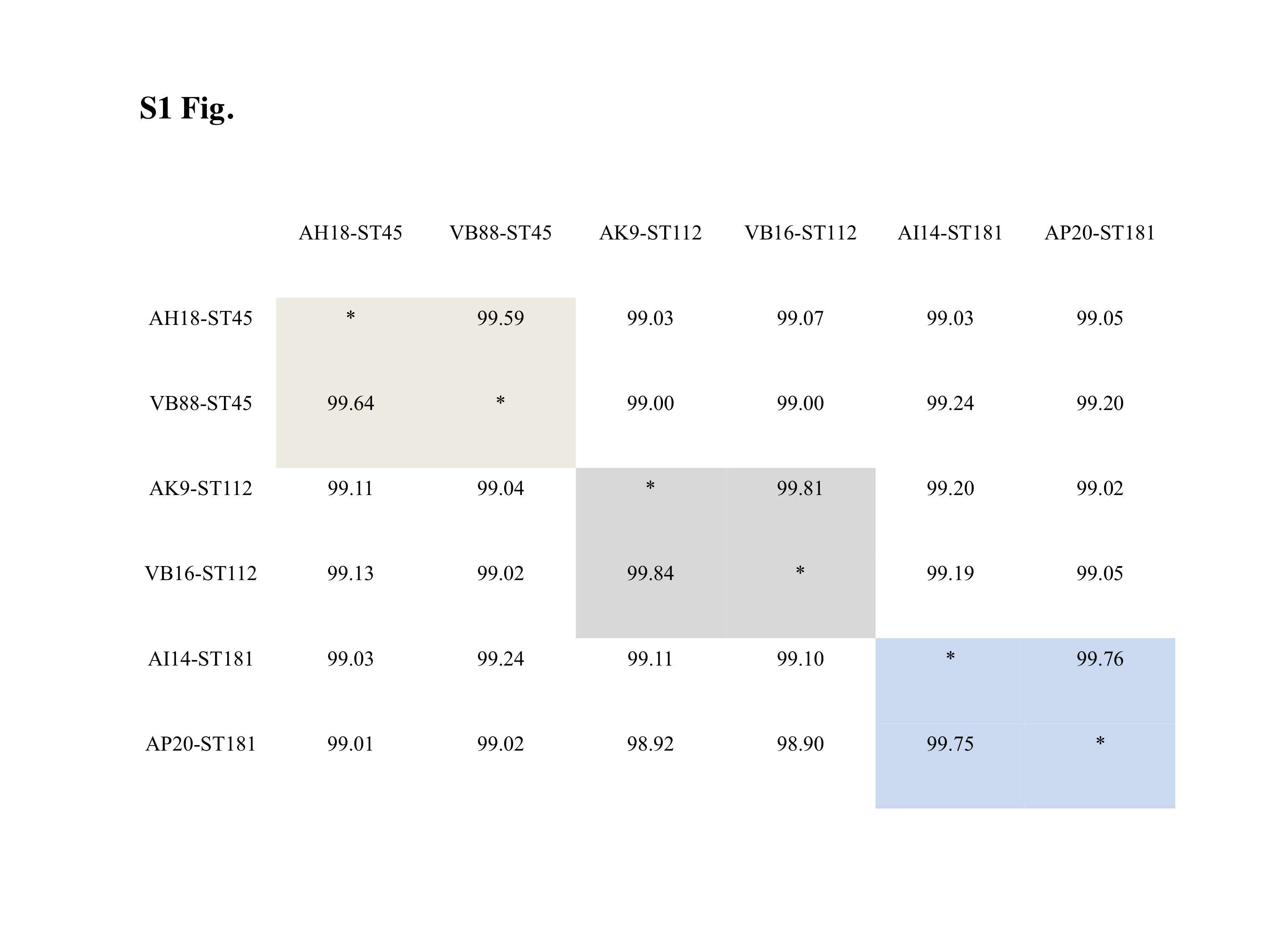

Supplement: S1 Fig — (TIFF) [file pone.0254382.s004.tiff]

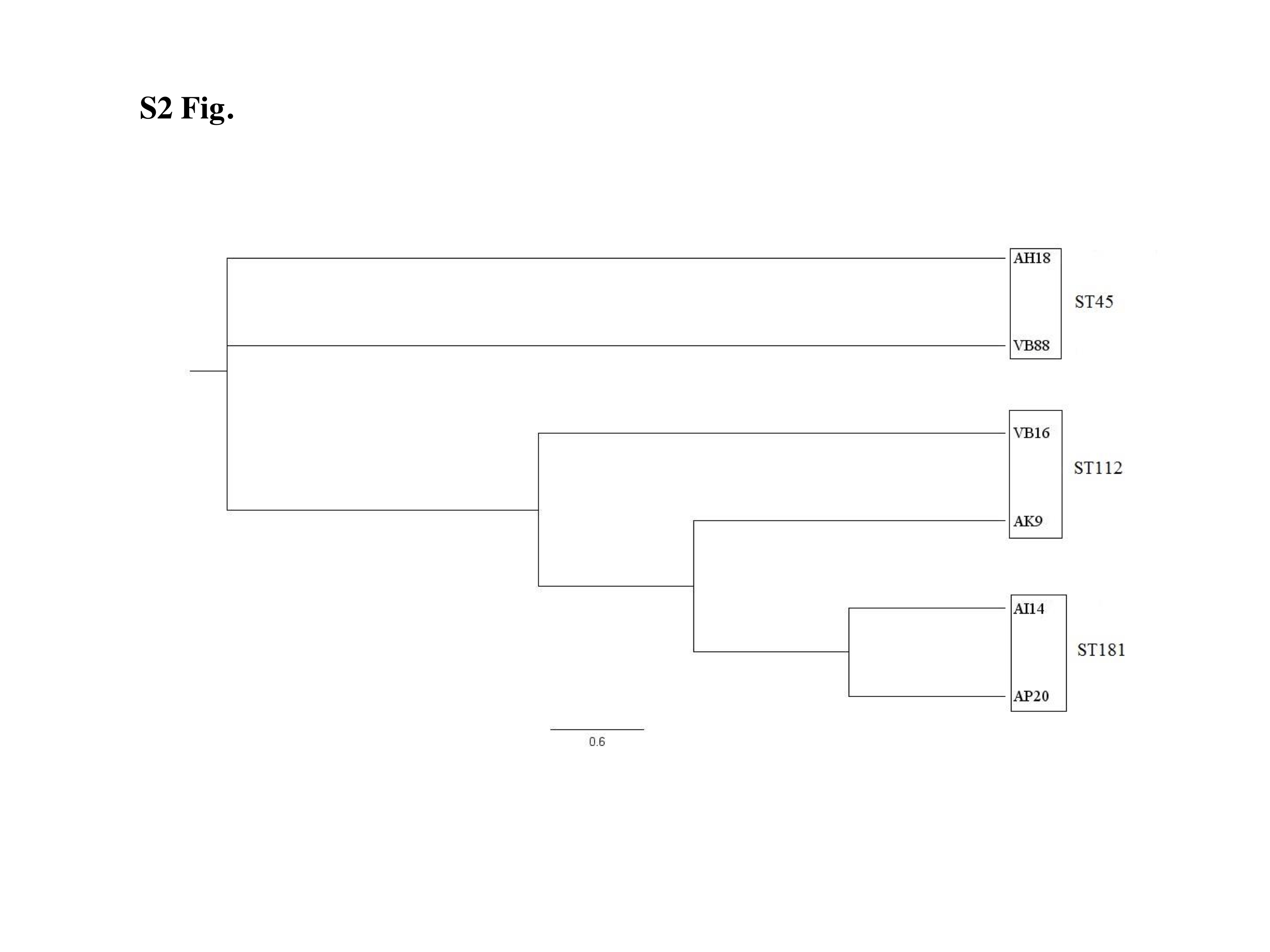

Supplement: S2 Fig — SNPs tree was constructed by CSIPhygeny 1.4. (TIFF) [file pone.0254382.s005.tiff]

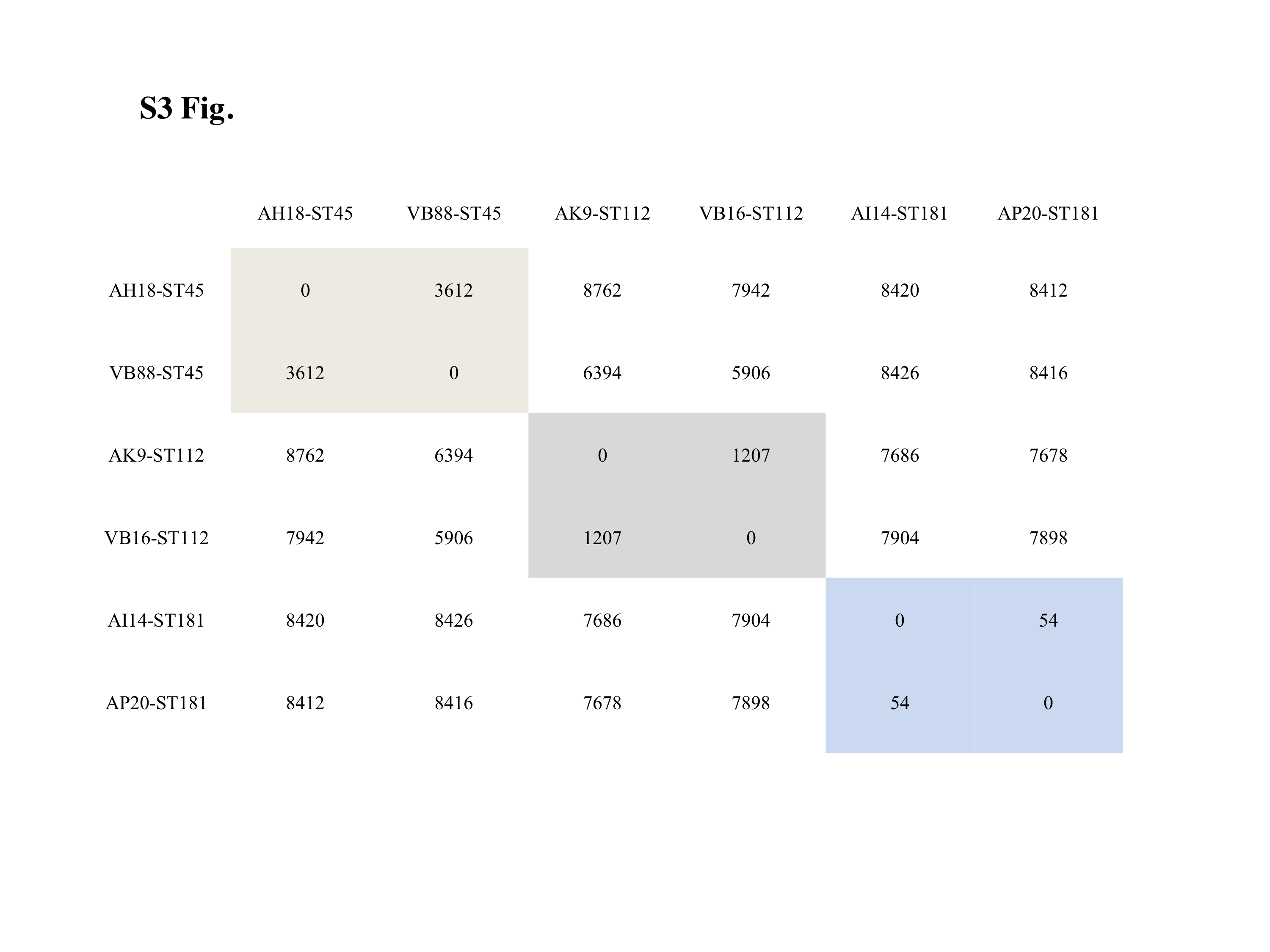

Supplement: S3 Fig — SNPs numbers were calculated by CSIPhygeny 1.4. Percentage of reference genome covered by all isolates: 81.9109943221414. Size of reference genome S. pseudintermedius NA45 was 2,841,212 bp. (TIFF) [file pone.0254382.s006.tiff]

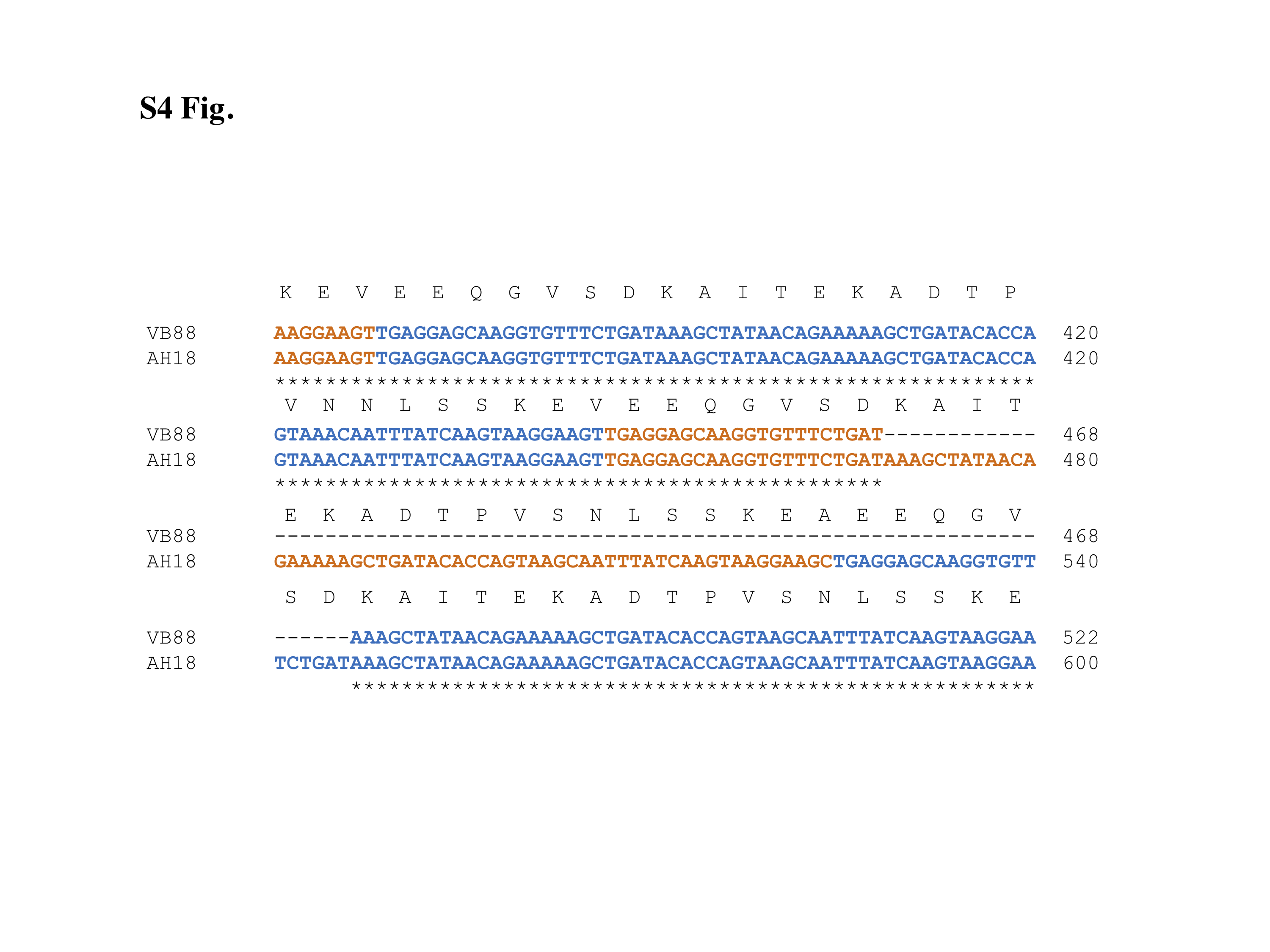

Supplement: S4 Fig — Pairwise alignment was performed in Genious v 10.1.3 using Clustal W defaults setting. (TIFF) [file pone.0254382.s007.tiff]

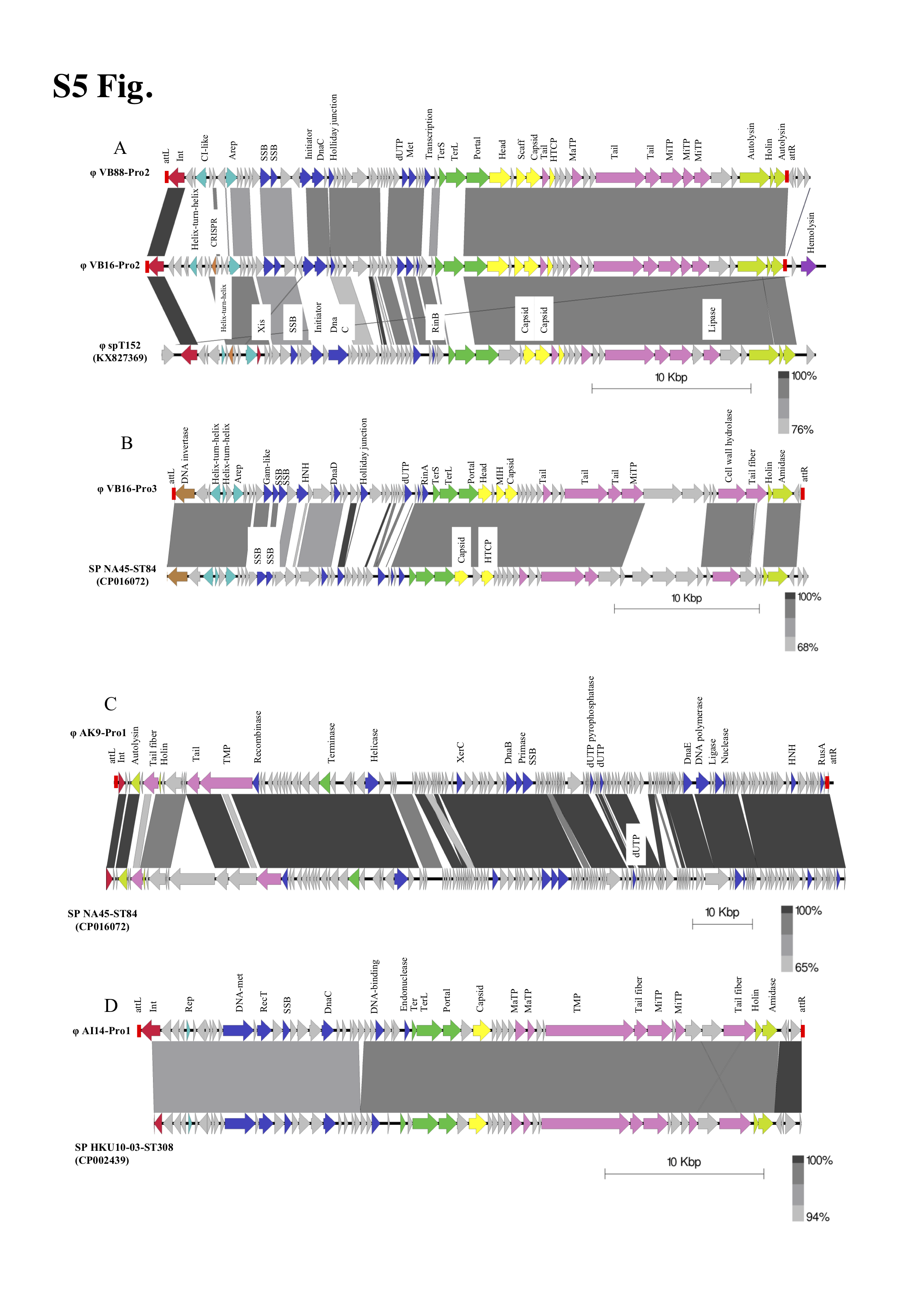

Supplement: S5 Fig — Comparison of phage modules of φ VB88-Pro2, φ VB16-Pro2, and φ spT152 (A), φ VB16-Pro3 and sequence of S. pseudintermedius NA45 (B), φ AK9-Pro1 and sequence of S. pseudintermedius NA45 (C), and φ AI14-Pro1 with sequence of S. pseudintermedius HKU10-03- ST308 (D). att, putative attachment site left (L) and right (R); Int, integrase; Helix-turn-helix, XRE family protein; CI-like; CI-like protein; Rep, phage repressor; Arep, anti-repressor; Xis, exisionase; HNH, HNH endonuclease; Gam-like, bacteriophage Mu Gam-like protein; SSB, single-stranded DNA- binding protein; DNA-met, DNA-cytosine methyltransferase; RecT, recombinational prophage- associated DNA repair protein RecT; dUTP, dUTPase; Met, methyltransferase; RinB, transcriptional regulator RinB; Initiator, phage replication initiation protein; DnaB, DNA replication protein DnaB; DnaC, DNA replication protein DnaC; DnaD, DNA replication protein DnaD; XerC,Tyrosine recombinase XerC; RusA, Holliday junction resolvase; TerS, terminase small subunit; TerL, terminase large subunit; HTCP, head-tail adaptor protein; MaTP, major tail protein; TMP, phage tail tape measure protein; Tail, phage tail protein; MiTP, minor tail protein; Scaff, scaffold protein. Figure was generated by Easyfig 2.1. (TIFF) [file pone.0254382.s008.tiff]
